# GAS reduced inflammatory responses in activated microglia by regulating the Ccr2/Akt/Gsk-3β pathway

**DOI:** 10.1186/s13041-025-01206-w

**Published:** 2025-05-06

**Authors:** Haolong Shi, Jinsha Shi, Zhao Wang, Hanjun Zuo, Tao Guo, Huixin Zheng, Rong Xiao, Xinglin Zhang, Shuhan Yang, Juanjuan Li

**Affiliations:** Department of Anatomy and Histology & Embryology, Faculty of Basic Medical Sciences, Kunming Medical University, 1168 West Chunrong Road, Kunming, Yunnan, 650500 PR China

**Keywords:** GAS, Microglia, Hypoxic-ischemic brain damage, Ccr2/Akt/Gsk-3β

## Abstract

Hypoxic-ischemic brain damage (HIBD) is a significant cause of neonatal death and neurological dysfunction. Following this injury, activated microglia can lead to a series of inflammatory responses. Gastrodin (GAS), a polyphenol extracted from the Chinese herbal medicine Gastrodia elata Blume, has demonstrated antioxidant and anti-inflammatory effects. This study investigated the neuroprotective impact of GAS in HIBD mice model and in BV2 cells subjected to oxygen-glucose deprivation (OGD) treatment. Expression of various members of the Ccr2/Akt/Gsk-3β, including *Ccl2*, Ccr2, Akt, p-Akt, Gsk-3β, p-Gsk-3β and inflammatory factors TNF-α and IL-1β in activated microglia was assessed by Western blotting, immunofluorescence, and qRT-PCR in HIBD in postnatal mice, and in OGD-induced BV2 microglia in vitro with or without GAS treatment. The present results showed that GAS effectively reduces the expression of *Ccl2* and Ccr2, increases the phosphorylation levels of Akt and Gsk-3β, and decreases the expression of the TNF-α and IL-1β. Additionally, we have shown that inhibition of Ccr2 by RS102895 increased the expression of p-Akt and p-Gsk-3β, and attenuate production of proinflammatory mediators in activated microglia. Of note, the expression of p-Akt, p-Gsk-3β, TNF-α and IL-1β remained unchanged after the combination of gastrodin and RS102895. Taken together, we conclude that GAS can play a protective role in reducing the neuroinflammatory response after HIBD. It is suggested that this is mainly through up-regulating the Akt/Gsk-3β signaling pathway via the Ccr2 receptor in the present experimental paradigm.

**Supplementary Information:**

The online version contains supplementary material available at 10.1186/s13041-025-01206-w.

## Introduction

Hypoxic-ischemic brain damage (HIBD) is a condition in newborns caused by various factors during the perinatal period, resulting in insufficient oxygen supply to the brain and ultimately leading to severe brain damage. It is a major contributor to neonatal mortality and neurological dysfunction [1]. Hypoxia can activate brain immune cells, such as microglia, neutrophils and lymphocytes, triggering the release of inflammatory factors and participation in neuroinflammation after brain injury [2]. Microglia, the resident immune cells of the central nervous system, become activated and exhibit phagocytic effects shortly after injury. They produce and release inflammatory factors, such as tumor necrosis factor α (TNF-α), interleukin-1β (IL-1β), interferon-γ (IFN-γ), interleukin-6 (IL-6), and inducible nitric oxide synthase (iNOS), through various pathways. These factors can disrupt the blood-brain barrier, allowing peripheral white blood cells to infiltrate the brain, thereby exacerbating inflammation and neuronal damage [3]. Research indicates an increased expression of activated microglia at the site of brain injury [4]. Furthermore, reducing microglial activation has been shown to alleviate brain injury [5]. Thus, inhibiting the activation of microglia may be an important strategy to mitigate neonatal HIBD injury.

Chemokines are small secreted proteins that attract and activate immune and non-immune cells both in vitro and in vivo [6]. With a molecular weight between 8 and 12 kDa, they recruit immune cells to the site of inflammation [7]. Among these, CC chemokine ligand 2 (Ccl2), also known as monocyte chemoattractant protein-1 (MCP-1), is a crucial chemokine that regulates monocyte/macrophage migration and infiltration. Ccl2 is primarily produced in the brain by astrocytes and resident microglia [8]. It interacts with its primary receptor, CC chemokine receptor 2 (Ccr2), a G-protein-coupled receptor. When Ccr2 binds to its ligand Ccl2, it activates downstream pathways including PKC, CaMKII, PI3K, protein kinase B (Akt), and ERK. These pathways are essential in processes such as cell migration, survival, transcriptional regulation and the release of nociceptive molecules [9]. Studies have shown significant increases in Ccl2 and Ccr2 expression levels after 6 h of cerebral ischemia and reperfusion in rats [10]. Notably, in mice lacking the Ccr2 receptor, cerebral edema, leukocyte infiltration, and the expression of inflammatory mediators in the ischemic hemisphere were significantly reduced after ischemia-reperfusion [11], implying a critical role of Ccl2/Ccr2 in the inflammatory response following brain injury. Additionally, Ccl2 induces the phosphorylation of Akt in astrocytes, which affects their survival [12]. In models of retinal inflammation, blocking Akt significantly reduced TNF-α-induced Ccl2 production, suggesting a close relationship between the downstream signal transduction of Ccl2/Ccr2 and Akt [13].

Akt plays a central role in cellular signaling downstream of growth factors, cytokines, and various cellular stimuli [14]. In the central nervous system (CNS), glycogen synthase kinase 3β (Gsk-3β), a serine-threonine kinase that is abundantly expressed in the brain, is involved in diverse cellular activities, including apoptosis and endogenous neurogenesis [15]. As a downstream target of Akt, Gsk-3β is phosphorylated by Akt at the serine 9 (Ser9) site, leading to the inhibition of its activity and a subsequent reduction in apoptosis [16]. Several studies suggest the involvement of the Akt/Gsk-3β pathway in neuronal apoptosis following hypoxic-ischemic brain injury and oxygen-glucose deprivation (OGD) injury in neonatal rats [17]. Additionally, research indicates the participation of the Akt/Gsk-3β pathway in neuroinflammation triggered by subarachnoid hemorrhage [16]. Therefore, it is essential to further explore the role of the Akt/Gsk-3β pathway in microglia-mediated inflammation after HIBD.

The current clinical treatment strategy for HIBD primarily involves hypothermia. While beneficial, therapeutic hypothermia does not provide complete neuroprotection. Survivors remain susceptible to neurodevelopmental abnormalities and the risk of mortality post-treatment [18]. This highlights the limitations in its efficacy. Gastrodin (GAS), an active compound derived from the traditional Chinese herb *Gastrodia elata*, exhibits anti-inflammatory, antioxidant, and immunomodulatory properties [19]. Research has shown that GAS exerts a protective effect on the survival of BV2 cells under hypoxic conditions [20]. In rat models, GAS significantly reduces histological injury caused by ischemia-reperfusion, decreases neuronal apoptosis, and lowers the expression of inflammatory and pro-apoptotic factors, thereby playing a neuroprotective role [21]. Additionally, GAS inhibits the activation of spinal microglia and reduces the expression of inflammatory factors through the chemokine CX3CL1 and its receptor CX3CR1 [22]. Furthermore, GAS can reduce ischemic damage in retinal ganglion cells via the PI3K/Akt/Nrf2 signaling pathway [23]. These studies underscore the potential therapeutic value of GAS; however, the precise mechanism underlying its neuroprotective effect requires further exploration.

This study aims to elucidate the neuroprotective potential of GAS in mitigating microglia-mediated inflammatory responses following HIBD via the Ccr2/Akt/Gsk-3β pathway.

## Materials and methods

### HIBD animal model

The research was conducted within an appropriate ethical framework, with experimental animals provided by Henan Skobes Biotechnology Co., Ltd. (License number: SCXK (Yu) 2020-0005). We used 54 C57BL/6J mice, 10 days old, weighing 3–5 g, of SPF grade. The mother mice were housed in the same cage within the IVC rodent feeding system, maintaining room temperature at 22 °C, with light cycles from 7 AM to 7 PM, and access to ample food and water. All experimental protocols were approved by Kunming Medical University to minimize animal use and suffering. Mice were randomly divided into sham group (*n* = 18), HIBD group (*n* = 18), and GAS-pretreated (HIBD + GAS) group (*n* = 18). In the HIBD group, the left common carotid artery was ligated following sevoflurane anesthesia, then placed in an anoxic chamber at 28–30 °C with 7-9% oxygen concentration for 40 min. The HIBD + GAS group followed the same procedure as the HIBD group, but mice were intraperitoneally injected with GAS at a concentration of 100 mg/kg three times: before surgery, immediately after hypoxia, and 12 h after hypoxia. GAS (purity 99.7%) was supplied by Kunming Pharmaceutical Co., Ltd. (Kunming, China). The corpus callosum of the mice was meticulously dissected. Subsequently, Western blotting, qRT-PCR, and immunofluorescence techniques were employed to detect the expression of various proteins and genes, including Ccl2, Ccr2, Akt, p-Akt, Gsk-3β, p-Gsk-3β, as well as the inflammatory factors TNF-α and IL-1β.

### BV2 microglia culture and treatments

BV2 cells were cultured in Dulbecco’s modified Eagle’s medium (DMEM) supplemented with 10% fetal bovine serum (FBS) in a 5% CO_2_ humidified incubator at 37℃. Upon reaching appropriate cell growth density, cells were seeded into six-well plates at a density of 6 × 10^5^ cells /ml. The experimental groups were as follows: Control, oxygen glucose deprivation (OGD), OGD + GAS (OGD + GAS), GAS (GAS). Groups containing GAS were pretreated with a concentration of 0.34 mM for 1 h, based on previous research results [19]. Then, the media for the OGD group was replaced with serum-free and glucose-free media and placed in anoxic chamber (95% N_2_, 5% CO_2_, temperature 37℃) for 2 h. To further investigate the effect of gastrodin on the Ccr2/Akt/Gsk-3β signaling, the Ccr2 antagonist RS102895 was used for intervention, The groups included: Control, RS102895, OGD, OGD + RS102895, OGD + RS102895 + GAS. Based on previous studies [24], before other treatments, the cells were pre-treated with RS102895 (Tocris, UK) at a concentration of 100 ng/ml for 1 h, followed by treatment with GAS at a concentration of 0.34 mM for 1 h. Subsequently, the corresponding groups were subjected to OGD treatment.

### RNA isolation and quantitative real time-PCR (qRT-PCR)

Total RNA was extracted from various brain tissues and cells using TRIzol reagent (Invitrogen, USA). cDNA was then synthesized using a first strand cDNA synthesis kit (NovoScript). This cDNA served as the template for the qPCR step in the qRT-PCR protocol. The reaction mixture (20 µL) was prepared according to the kit’s instructions, consisting of 10 µL 2× NovoStart^®^ SYBR qPCR Supermix Plus, 0.8 µL upstream primer, 0.8 µL downstream primer, 2 µL cDNA template, 0.4 µL ROX Reference Dye II, and 6 µL RNase-free ddH_2_O. Using β-actin as the internal standard, the relative mRNA expression was calculated using the 2^−ΔΔCt^ method. The experiment was performed independently at least three times. The primer sequences are as follows: Ccl2 (sense: CAACTCTCACTGAAGCCAG, antisense: TTAACTGCATCTGGCTGAG); Ccr2 (sense: CCTGTAAAGACCTCAGCCCA, antisense: GGCACTGCTATCTCAGGCTT); β-actin (sense: GCTATGTTGCTCTAGACTTCG, antisense: GGATTCCATACCCAAGAAGG).

### Western blotting

Tissue and cell proteins were extracted using RIPA buffer, with a specific focus on the corpus callosum due to its abundance of microglia in postnatal rats. The tissue was then thoroughly ground on ice, and the supernatant was centrifuged after standing at a low temperature for 10 min. BV2 cells were washed twice with PBS, and then RIPA lysis buffer and a protease inhibitor mixture were added. The mixture was allowed to sit before the cells were scraped with a scraper, left to stand for 10 min, and then the supernatant was centrifuged. The concentration of the protein samples was determined using the BCA method. The proteins were separated using 10% sodium dodecyl sulfate-polyacrylamide gel electrophoresis (SDS-PAGE). Protein bands were transferred onto polyvinylidene fluoride (PVDF) membranes and blocked with skim milk. They were incubated overnight at 4 °C with the following antibodies: anti-Ccr2 (1:2000; Rabbit polyclonal; ORIGENE; TA350635), Akt (1:2000; Mouse monoclonal; ORIGENE; TA504230), p-Akt (1:2000; Rabbit polyclonal; ORIGENE; TA325218), anti-Gsk3 beta (1:1000; Rabbit polyclonal; ORIGENE; TA367586), anti-phosphorylated (p)-Gsk3 beta (1:2000; Rabbit polyclonal; ORIGENE; TA376823), anti-TNF alpha (1:500; Rabbit polyclonal; Affinity; AF7014), anti-IL-1β (1:2000; Rabbit polyclonal; ORIGENE; TA319453), anti-beta actin (1:2000; Mouse monoclonal; ORIGENE; TA811000), and anti-GAPDH (1:2000; Mouse monoclonal; ORIGENE; TA802519). The next day, membranes were incubated for 1 h with anti-mouse IgG or anti-rabbit IgG coupled with horseradish peroxidase (HRP). After washing, the protein bands were detected using a chemiluminescence imager. All experiments were repeated at least three times, and the data were analyzed using Image J software.

### Immunofluorescence labeling of hypoxia-ischemia brain

Mice were deeply anesthetized with sevoflurane and then sacrificed by cardiac perfusion with 2% paraformaldehyde. Following brain extraction, dehydration was performed and the tissue was then paraffin-embedded. Coronal sections of 7 μm thickness were obtained and rehydrated using a 0.01 M citric acid buffer solution. Subsequently, the sections were blocked with 10% goat serum and then incubated overnight at 4℃ in a humidified box with primary antibodies: anti-Ccr2 (1:100), p-Akt (1:200), and anti-phosphorylated (p)-Gsk3 beta (1:200). Following primary antibody incubation, the sections were incubated with the secondary antibodies for 1 h at room temperature. The secondary antibodies were FITC-conjugated lectin (1:200; SIGMA; Cat. No. L0401), Cy3-conjugated goat anti-mouse IgG (1:200; Proteintech; Cat. No. SA00009-1) and Cy3-conjugated goat anti-rabbit IgG (1:200; Proteintech; Cat. No. SA00009-2). Finally, the sections were sealed with a fluorescent sealer containing 4’, 6-diamidino-2-phenylindole (DAPI). Co-localization was verified using confocal microscopy.

### Immunofluorescence labeling of BV2 microglia

The cells were inoculated at a density of 1.2 × 10^4^ cells per well in a 24-well plate and cultured for 24 h. Following this, the cells were fixed with 4% paraformaldehyde for 20 min and blocked with 10% goat serum at room temperature for 2 h. They were then incubated overnight at 4°C with primary antibodies: anti-Ccr2, Akt, p-Akt, anti-Gsk3 beta and anti-phosphorylated (p)-Gsk3 beta. Following washing steps with PBS, the cells were incubated with the secondary antibodies for 1 h at room temperature. The secondary antibodies were FITC-conjugated lectin, Cy3-conjugated goat anti-mouse IgG, and Cy3-conjugated goat anti-rabbit IgG. Finally, the cells were sealed with a fluorescent sealer containing DAPI. Fluorescence microscopy was used to capture images, and the immunofluorescence intensity was quantified using Image J software.

### Cell viability assay of BV2 microglia

The 96-well plate was inoculated with a cell suspension (5,000 cells per well) and cultured for 24 h. The medium was then aspirated and the wells were washed twice with PBS. Different concentrations of RS102895 (0 ~ 200 ng/ml) were added to the corresponding wells and the cells were treated for 1 h. Afterward, the medium was aspirated, the wells were washed twice with PBS, and then the basic medium containing 20 µl of CCK-8 solution was added. The absorbance at 450 nm was measured using a microplate reader after 3 h of incubation. The experiment was repeated three times.

### Statistical analysis

Statistical analysis of all experimental results was conducted using GragpahPad Prism software. The data presented in the bar chart were expressed as the mean ± standard deviation (SD) derived from a three independent experiments. One-way analysis of variance (One-WayANOVA) was used for comparison between groups, and *P* < 0.05 indicated statistically significant differences. *P < 0.05; **P < 0.01, ***P < 0.001.

## Results

**GAS inhibits the mRNA expression of** ***Ccl2*** **and** ***Ccr2*** **in HIBD mice**.

**Fig. 1:**
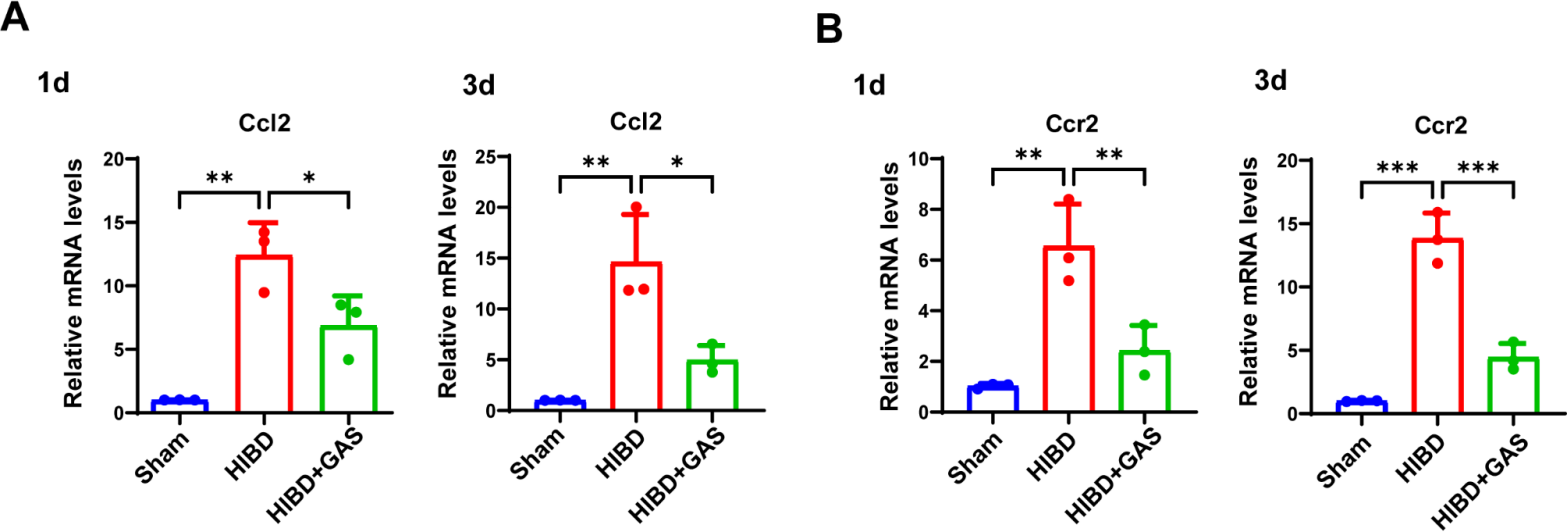
GAS decreased the mRNA levels of *Ccl2* and Ccr2 in the mice following HIBD. **A** The expression of *Ccl2* mRNA at 1 day and 3 days after HIBD; **B** The expression of *Ccr2* mRNA at 1 day and 3 days after HIBD. *n* = 3, *P < 0.05, **P < 0.01, **P < 0.001

The qRT-PCR results showed that *Ccl2* (1 day *p* = 0.001, 3 day *p* = 0.003) and *Ccr2* (1 day *p* = 0.002, 3 day p = < 0.001) mRNA in the ischemic corpus callosum of HIBD mice were significantly higher than that in the sham group at both 1 day and 3 days after injury. Furthermore, GAS treatment significantly decreased *Ccl2* (1 day *p* = 0.034, 3 day *p* = 0.014) and *Ccr2* mRNA levels (1 day *p* = 0.008, 3 day p = < 0.001) (Fig. 1).

### GAS decreases the expression of Ccr2 and increased the phosphorylation of Akt and Gsk-3β in HIBD mice

**Fig. 2:**
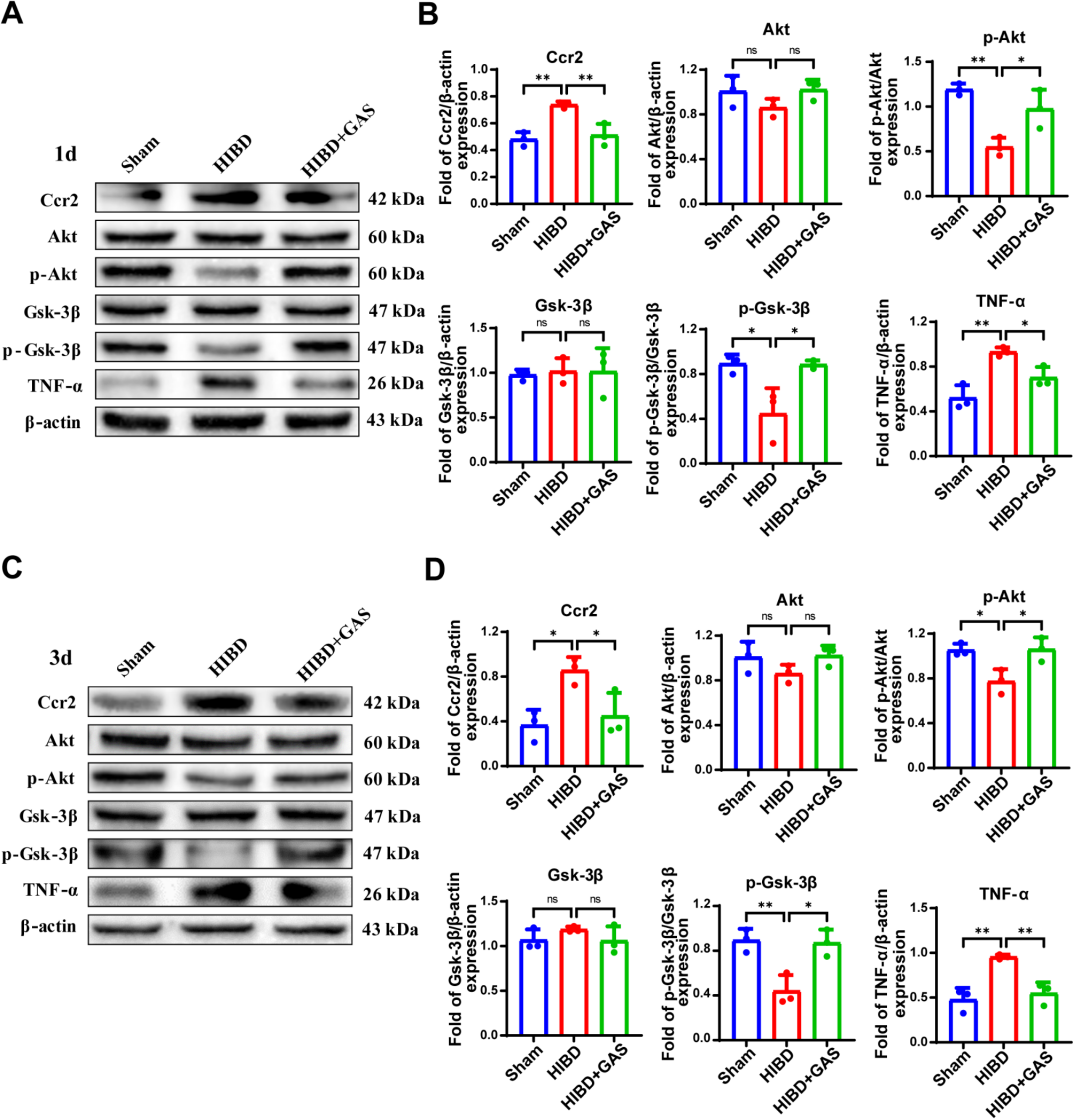
GAS inhibits Ccr2 and TNF-α expression, while increased of Akt and Gsk-3β phosphorylation. **A** The Ccr2, Akt, Gsk-3β and TNF-α expression changes on 1 day of HIBD detected by Western blotting. **B** The protein content in the brain tissue of mice at 1 day after HIBD was analyzed. **C** The Ccr2, Akt, Gsk-3β and TNF-α expression changes on 3 days of HIBD detected by Western blotting. **D** The protein content in the brain tissue of mice at 3 days after HIBD was analyzed. *n* = 3, *P < 0.05, **P < 0.01

Western blotting results showed that, compared with the sham group, the expression levels of Ccr2 and TNF-α in the ischemic corpus callosum of HIBD mice were significantly increased at both 1 day (*p* = 0.004 and *p* = 0.003, respectively) and 3 days (*p* = 0.021 and *p* = 0.003, respectively) after injury, while the phosphorylation levels of Akt (1 day *p* = 0.004, 3 day *p* = 0.03) and Gsk-3β (1 day *p* = 0.020, 3 day *p* = 0.009) were significantly decreased. However, Ccr2 (1 day *p* = 0.008, 3 day *p* = 0.046) and TNF-α (1 day *p* = 0.043, 3 day *p* = 0.008) expression levels were significantly reduced, and the phosphorylation levels of Akt (1 day *p* = 0.025, 3 day *p* = 0.023) and Gsk-3β (1 day *p* = 0.021, 3 day *p* = 0.012) were significantly elevated after GAS treatment (Fig. 2).

**Fig. 3:**
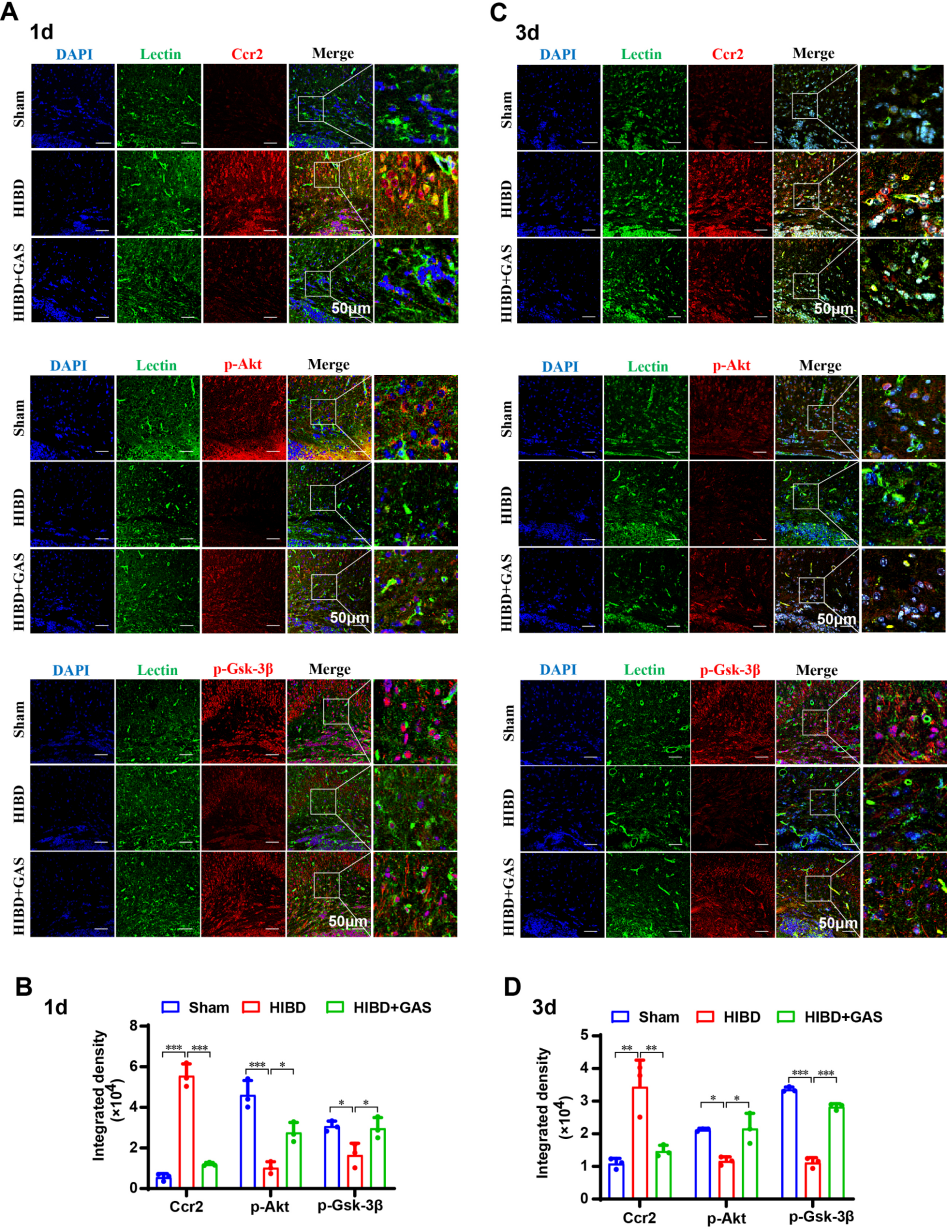
GAS inhibits Ccr2 expression and increased the Akt and Gsk-3β phosphorylation in HIBD mice. **A** Confocal microscope images show the co-localization of Ccr2, p-Akt, p-Gsk-3β (red) in Lectin + microglia cells (green) at 1 day after HIBD. **B** Presents the average immunofluorescence values of Ccr2, p-Akt, and p-Gsk-3β at 1 day after HIBD. **C** Confocal microscope images show the co-localization of Ccr2, p-Akt, p-Gsk-3β (red) in Lectin + microglia cells (green) at 3 days after HIBD. **D** Presents the average immunofluorescence values of Ccr2, p-Akt, and p-Gsk-3β at 3 days after HIBD. *n* = 3, *P < 0.05, **P < 0.01, ***P < 0.001. Scale: 50 μm

The immunofluorescence results demonstrated that compared with the sham group, after HIBD, the fluorescence intensity of Ccr2 and its co-expression with the microglial marker lectin in the callosum region were significantly increased (1 day *p* < 0.001, 3 day *p* = 0.003), while the p-Akt (1 day *p* < 0.001, 3 day *p* = 0.013) and p-Gsk-3β (1 day *p* = 0.025, 3 day *p* < 0.001) were significantly decreased. Following GAS treatment, the fluorescence intensity of Ccr2 was significantly reduced (1 day *p* < 0.001, 3 day *p* = 0.006), whereas the p-Akt (1 day *p* = 0.017, 3 day *p* = 0.011) and p-Gsk-3β (1 day *p* < 0 0.001, 3 day *p* < 0.001) were significantly elevated (Fig. 3).

### GAS decreases the expression of *Ccl2* and Ccr2, while increasing the phosphorylation levels of Akt and Gsk-3β in BV2 microglia following OGD

**Fig. 4:**
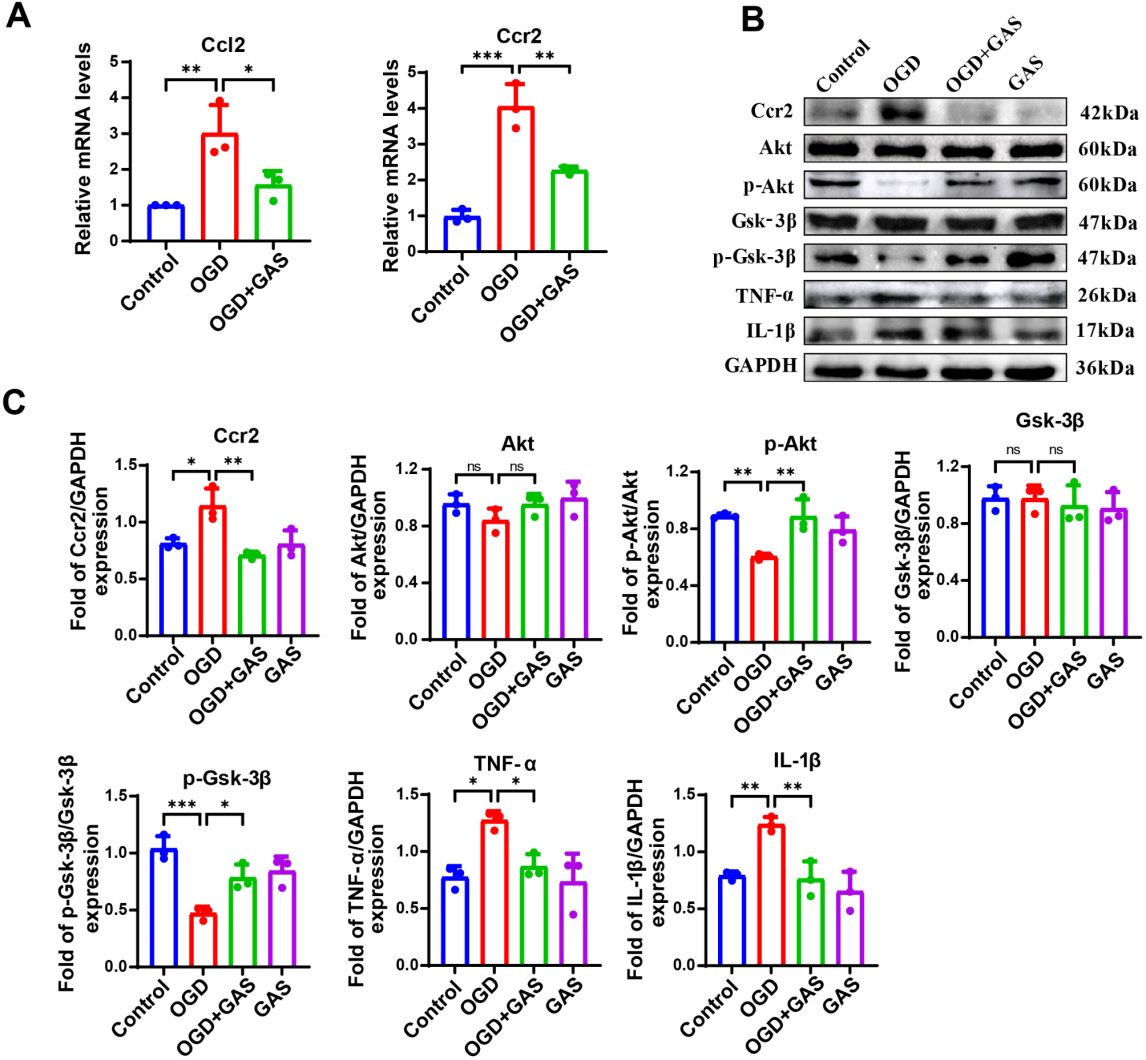
GAS decreases the mRNA level of *Ccl2* and *Ccr2* and activated the Ccr2/Akt/Gsk-3β pathway after OGD in BV2 cells. **A** Compared with the control group, the mRNA levels of *Ccl2* and *Ccr2* were significantly increased in the OGD group and decreased after GAS treatment. **B** Compared with the control group, the protein levels of Ccr2, TNF-α, and IL-1β in the OGD group were significantly increased and decreased after GAS treatment. The phosphorylation levels of Akt and Gsk-3β were significantly decreased in the OGD group and increased after GAS treatment. **C** The protein content in BV2 cells. *n* = 3, *P < 0.05, **P < 0.01, ***P < 0.001

The qRT-PCR results showed that mRNA levels of *Ccl2* (*p* = 0.007) and *Ccr2* (*p* < 0.001) in the OGD group were significantly higher than those in the control group. Notably, GAS treatment led to a significant reduction in mRNA levels of *Ccl2* (*p* = 0.031) and *Ccr2* (*p* = 0.003) (Fig. 4A).

In BV2 microglia, Western blotting analysis demonstrated a significant increase in the expression of Ccr2 (*p* = 0.018), TNF-α (*p* = 0.014), and IL-1β (*p* = 0.008), along with a significant decrease in the phosphorylation levels of Akt (*p* = 0.009) and Gsk-3β (*p* < 0.001) after OGD, compared with the control group. Remarkably, GAS treatment significantly decreased the expression of Ccr2 (*p* = 0.004), TNF-α (*p* = 0.041), and IL-1β (*p* = 0.006), while significantly increasing the phosphorylation levels of Akt (*p* = 0.010) and Gsk-3β (*p* = 0.014) (Fig. 4B, C).

**Fig. 5:**
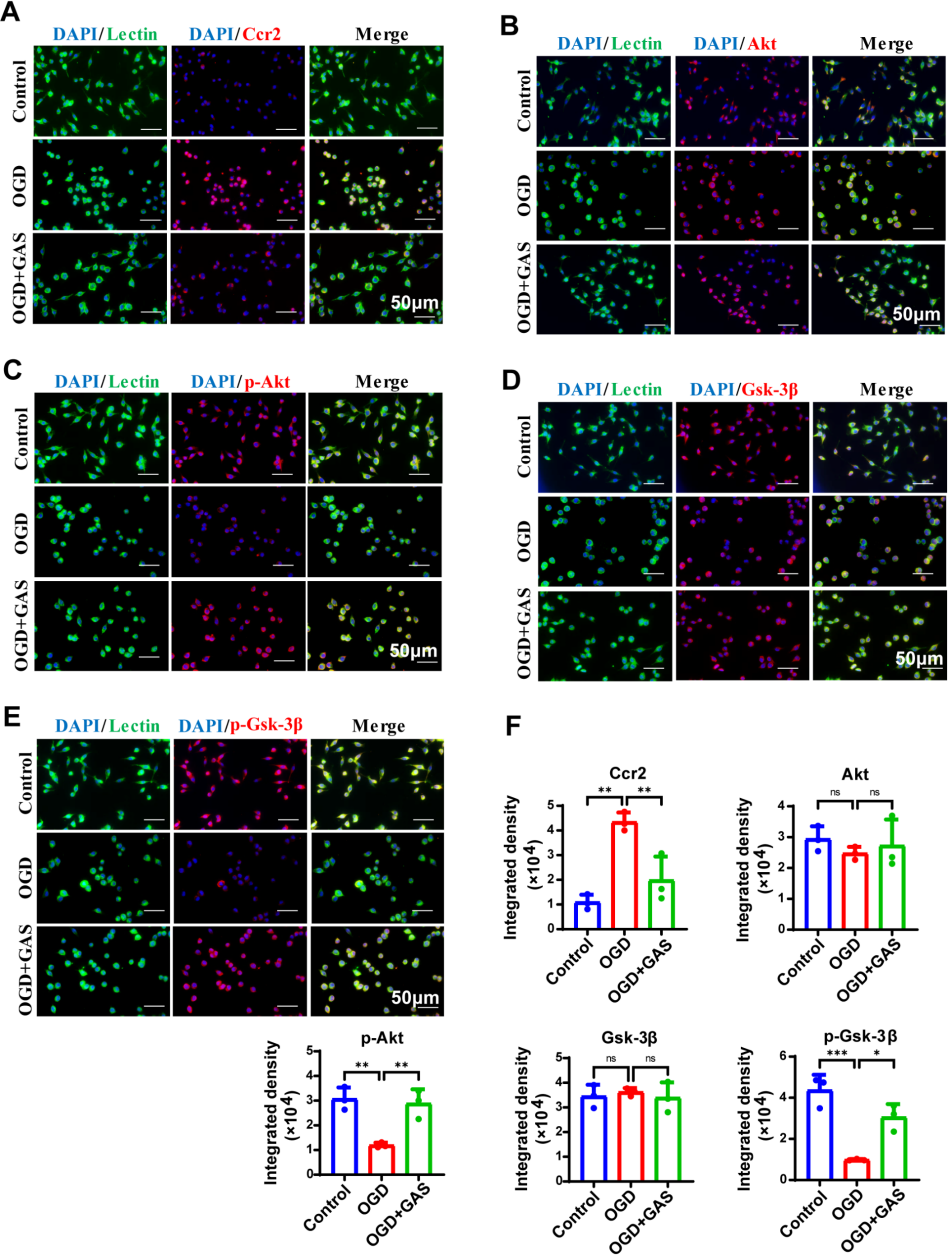
GAS activates the Ccr2/Akt/Gsk-3β pathway in OGD-induced BV2 cells. **A-E** Fluorescence images showed that compared with the control group, the levels of Ccr2 (red) in Lectin + microglia (green) were significantly increased, while the levels of p-Akt (red) and p-Gsk-3β (red) were significantly decreased in the OGD group. After GAS treatment, the expression of Ccr2 decreased, and the levels of p-Akt and p-Gsk-3β increased. DAPI (blue) indicates the nucleus. **F** Presents the average immunofluorescence values of Ccr2, Akt, p-Akt, Gsk-3β, and p-Gsk-3β. *n* = 3, *P < 0.05; **P < 0.01, ***P < 0.001. Scale: 50 μm

Immunofluorescence staining revealed that, compared with the control group, activated BV2 microglia significantly enhanced positive expression of Ccr2 (*p* = 0.002), while the positive expression of p-Akt (*p* = 0.004) and p-Gsk-3β (*p* < 0.001) was significantly weakened. In contrast, after GAS treatment, the fluorescence intensity of Ccr2 was significantly reduced (*p* = 0.008), whereas the fluorescence intensities of p-Akt (*p* = 0.007) and p-Gsk-3β (*p* = 0.011) were significantly increased (Fig. 5).

### GAS alleviates inflammation in OGD-induced BV2 microglia via the Ccr2/Akt/Gsk-3β signaling pathway

**Fig. 6:**
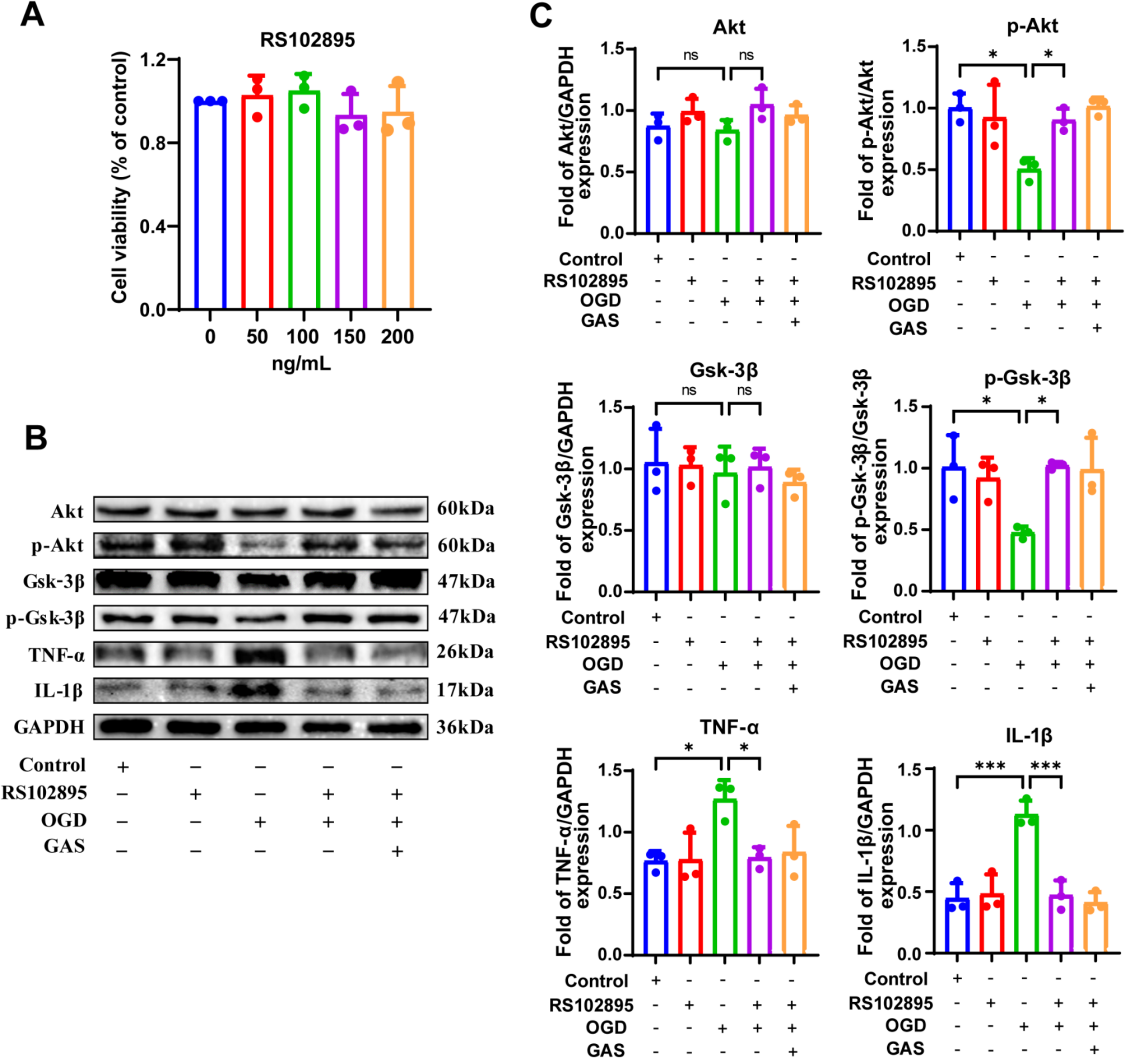
GAS inhibits the expression of inflammatory factors TNF-α and IL-1β through the Ccr2/Akt/Gsk-3β pathway. **A** RS102895 did not cause significant BV2 cell death in the 0 ~ 200 ng/mL range by CCK8. **B** Western blotting showed that the phosphorylation levels of Akt and Gsk-3β were significantly increased and the expression levels of TNF-α and IL-1β were significantly decreased in the OGD + RS102895 group compared with the OGD group. There were no significant differences in the expression of p-Akt, p-Gsk-3β, TNF-α, and IL-1β in the OGD + RS102895 + GAS group compared with the OGD + RS102895 group. **C** Presents the protein content in BV2 cells. *n* = 3, *P < 0.05, ***P < 0.001

CCK-8 assay results demonstrated that Ccr2 antagonist RS102895 did not cause significant BV2 cell death within the 0 ~ 200 ng/mL range (Fig. 6A). Western blotting analysis showed that RS102895 significantly increased the phosphorylation levels of Akt (*p* = 0.047) and Gsk-3β (*p* = 0.028) and decreased the expressions of TNF-α (*p* = 0.032) and IL-1β (*p* < 0.001) compared to the OGD group, indicating that Ccr2 plays a critical role in regulating the Akt/Gsk-3β pathway. It is worth noting that no significant differences in the expression of p-Akt, p-Gsk-3β, TNF-α and IL-1β were observed between the RS102895 treatment group and the RS102895 + GAS treatment group in OGD-induced BV2 cells (Fig. 6B, C). These results suggest that GAS reduces the expression of inflammatory cytokines TNF-α and IL-1β after OGD treatment, and this effect is mediated through the Ccr2/Akt/Gsk-3β pathway.

## Disscussion

HIBD is a major contributor to neonatal mortality and neurological dysfunction. Numerous studies have underscored the critical role of inflammation in the progression of HIBD. Inadequate management of the acute inflammatory phase can result in chronic inflammation, adversely affecting brain development [25]. Microglia, as a resident immune cell of the central nervous system, have play an essential role in maintaining brain homeostasis [26]. Following hypoxic-ischemic brain injury, microglia are rapidly activated, releasing a substantial amount of pro-inflammatory factors. These factors, together with activated brain immune cells and inflammatory mediators released by infiltrating blood cells, contribute to neuroinflammation, further exacerbating the injury [27]. Therefore, targeting the reduction of microglia-mediated neuroinflammation is considered a promising therapeutic strategy for treating neurodegenerative diseases, including HIBD.

Activated microglia release various factors, including cytokines, chemokines, and nitric oxide (NO), which contribute to the inflammatory response [28]. Ccl2, a member of the monocyte chemokine protein family, induces monocyte infiltration and mediates inflammation by binding to its receptor Ccr2. The Ccl2/Ccr2 axis plays a crucial role in transmitting neuroinflammatory signals between different cell types within the central nervous system [29]. In neonatal mice, the corpus callosum is the region where microglia predominantly aggregate, and it exhibits the most pronounced pathological changes following injury [30]. Based on these observations, corpus callosum was chosen to study. Our experiments showed that the levels of Ccl2 mRNA, as well as the protein expression of Ccr2 and the pro-inflammatory mediator TNF-α, were significantly increased in the corpus callosum on the injured side at 1 day and 3 days after HIBD in neonatal mice. Meanwhile, in vitro results indicated that *Ccl2* mRNA and Ccr2 protein expression levels were significantly elevated in OGD-induced BV2 microglia coupled with a significant upregulation in inflammatory cytokines TNF-α and IL-1β. These findings suggest that the activation of the Ccl2/Ccr2 axis following HIBD promotes the expression of proinflammatory mediators including TNF-α and IL-1β, contributing to microglia-mediated neuroinflammation after hypoxic-ischemic brain injury. However, the mechanism by which the Ccl2/Ccr2 axis promotes the inflammatory response in HIBD remains unclear.

When Ccl2 binds to Ccr2 it can activate various intracellular G protein-mediated signaling pathways, especially PI3K/Akt/ERK/NF-κB [31]. Activation of this pathway leads to the mobilization of transcription factors and genes associated with cytokine production, cell growth and differentiation, and inflammation [32], underscoring Akt is a promising target for Ccr2-mediated inflammation.

The Akt/Gsk-3β pathway is a critical signaling pathway for anti-apoptosis and neuroprotection [33]. Previous studies have highlighted the anti-inflammatory role of activating the Akt/Gsk-3β-Nrf2 signaling axis in BV2 cells [34]. Our study shows that following HIBD in mice and OGD in BV2 cells, the phosphorylation levels of Akt and Gsk-3β were significantly decreased, while TNF-α and IL-1β expression levels were significantly increased. This suggests that the Akt/Gsk-3β pathway is downregulated in microglia after hypoxic-ischemic brain injury, promoting the expression of inflammatory factors TNF-α and IL-1β and exacerbating the inflammatory response. Furthermore, after blocking Ccr2 with RS102895, the OGD + RS102895 group exhibited a significant increase in Akt and Gsk-3β phosphorylation levels compared to the OGD group, alongside a decrease in TNF-α and IL-1β expression. This indicates that Ccr2 may inhibit the Akt/Gsk-3β signaling pathway, upregulating the expression of inflammatory factors TNF-α and IL-1β, and contributing to the neuroinflammatory response triggered by activated microglia following HIBD injury.

Currently, hypothermia therapy is the primary clinical treatment for HIBD. However, due to its limitations, there is an urgent need to discover new therapeutic approaches. GAS, a bioactive compound extracted from the traditional Chinese medicine Gastrodia, has demonstrated beneficial effects on various neurological and mental disorders [20]. Recent studies have shown that GAS exerts anti-inflammatory, anti-apoptotic, and antioxidant effects through the PI3K/Akt-Sirt3 signaling pathway [35]. In this study, we found that GAS significantly reduced *Ccl2* mRNA levels and Ccr2 protein expression, upregulated the phosphorylation levels of Akt and Gsk-3β, and markedly decreased the expression of inflammatory factors TNF-α and IL-1β in activated microglia in the corpus callosum of HIBD mice, and in OGD inducecd BV2 microglia. These findings suggest that GAS reduces the microglia-mediated inflammatory response activated after HIBD and modulates the expression of proteins related to the Ccr2/Akt/Gsk-3β signaling pathway. It is therefore tempting to speculate that the anti-inflammatory effect of GAS on microglia during HIBD was related to Ccr2/Akt/Gsk-3β signaling pathway. The present results demonstrated that inhibition of Ccr2 increased the Akt and Gsk-3β phosphorylation levels and decreased expression levels of TNF-α and IL-1β in OGD-induced BV2 microglia. More importantly, the phosphorylation levels of Akt and Gsk-3β and proinflammatory mediators TNF-α and IL-1β expression was unobvious changes when Ccr2 inhibitor was administered along with GAS in activated BV2 microglia. These results suggest that GAS may exert its neuroprotective effects by activating the Akt/Gsk-3β pathway through the Ccr2 receptor, thereby inhibiting the expression of inflammatory factors TNF-α and IL-1β.

In conclusion, the present results have shown that GAS exerts a neuroprotective effect against HIBD-induced brain injury. Through in vivo and in vitro experiments, we found that GAS could inhibit the expression of proinflammatory mediators and modulate the expression of proteins related to the Ccr2/Akt/Gsk-3β signaling pathway in HIBD mice and OGD-induced activated microglia. Additionally, Similar to the GAS, the Ccr2 receptor antagonist RS102895 activated the Akt/Gsk-3β signaling pathway. However, the combination treatment of RS102895 and GAS had no significant impact on the Akt/Gsk-3β pathway or the expression of proinflammatory mediators compared to the RS102895 treatment in OGD-induced BV2 cells. These findings suggest that GAS may play a neuroprotective role by activating the Akt/Gsk-3β signaling pathway through the Ccr2 receptor and inhibiting microglia-mediated inflammatory responses. This discovery offers a novel therapeutic approach for the treatment of HIBD.

In this study, the mechanism by which gastrodin regulates microglia-mediated neuroinflammation through the Ccr2/Akt/Gsk-3β pathway was investigated solely using the Ccr2 antagonist RS102895, and the exploration of molecular mechanisms remains insufficiently comprehensive. In future research, we plan to employ microglial Ccr2 knockout (Ccr2^-/-^) mice to further investigate the effects of gastrodin on Ccr2^-/-^ HIBD mice through in vivo experiments. This approach aims to elucidate the mechanistic role of gastrodin in targeting Ccr2 to modulate inflammatory responses via the Akt/Gsk-3β signaling pathway.

## Electronic supplementary material

Below is the link to the electronic supplementary material.

Supplementary Material 1

Supplementary Material 2

## Data Availability

No datasets were generated or analysed during the current study.

## Abbreviations

Akt: Protein kinase B
Ccl2: CC chemokine ligand 2
Ccr2: CC chemokine receptor 2
RS102895: Ccr2 inhibition RS102895
CNS: Central nervous system
DAPI: 4’, 6-diamidino-2-phenylindole
DMEM: Dulbecco’s modified Eagle’s medium
FBS: Fetal bovine serum
GAS: Gastrodin
Gsk-3β: Glycogen synthase kinase 3β
HIBD: Hypoxic-ischemic brain damage
IFN-γ: Interferon-γ
IL-1β: Interleukin-1β
IL-6: Interleukin-6
iNOS: Inducible nitric oxide synthase
MCP-1: Monocyte chemoattractant protein-1
OGD: Oxygen-glucose deprivation
PVDF: Protein bands were transferred onto polyvinylidene fluoride
SDS-PAGE: Sodium dodecyl sulfate-polyacrylamide gel electrophoresis
TNF-α: Tumor necrosis factor α
