# Supplementary material for: GAS reduced inflammatory responses in activated microglia by regulating the Ccr2/Akt/Gsk-3β pathway
Source: Mol Brain. 2025 May 6;18:40. doi: 10.1186/s13041-025-01206-w (PMC12057146; doi:10.1186/s13041-025-01206-w)
Supplement: Supplementary file 2 — Supplementary Material 2 [file 13041_2025_1206_MOESM2_ESM.pptx]

## Slide 1
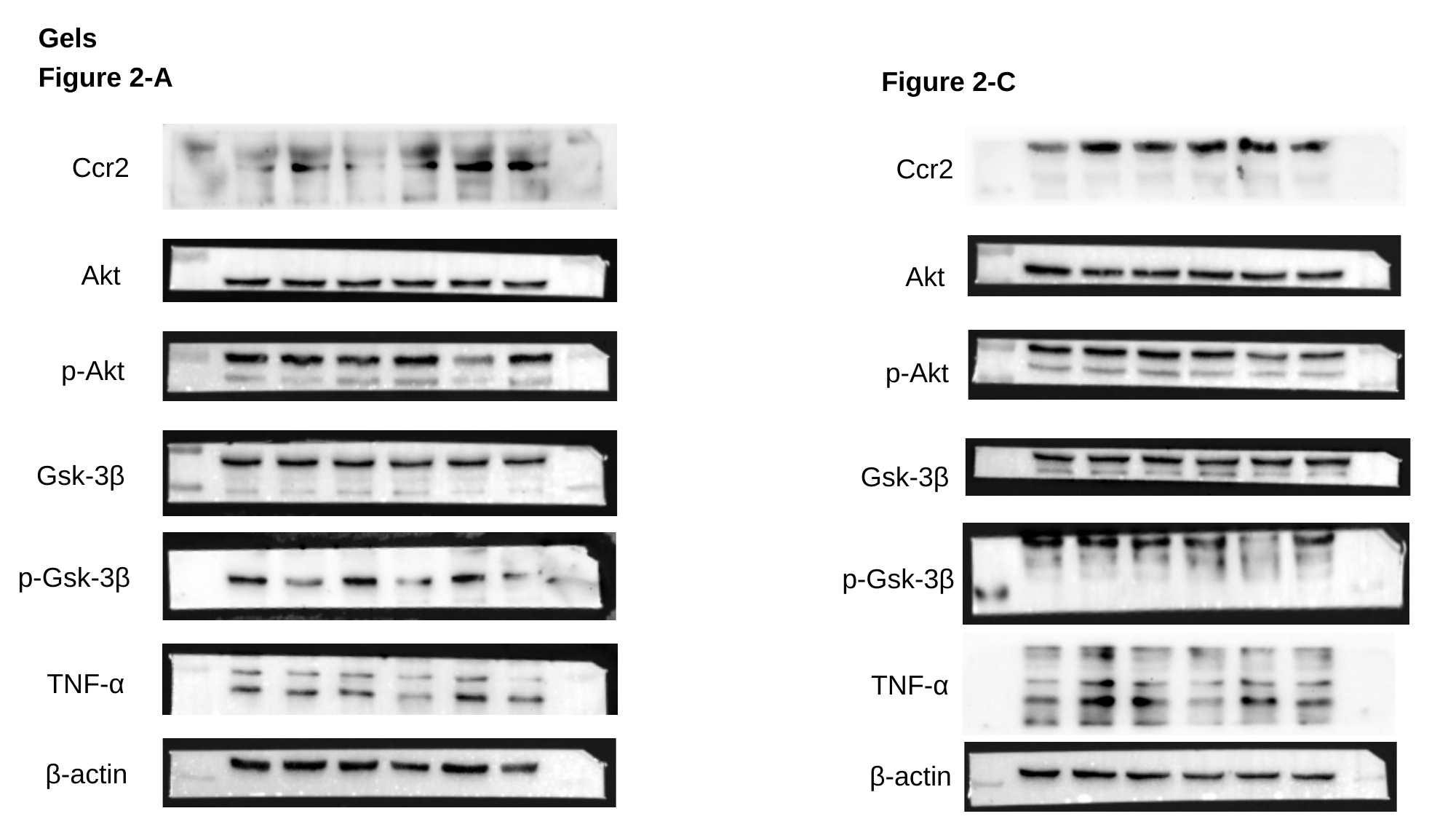

Gels
Figure 2-A
Figure 2-C
Ccr2
Ccr2
Akt
Akt
p-Akt
p-Akt
Gsk-3β
Gsk-3β
p-Gsk-3β
p-Gsk-3β
TNF-α
TNF-α
β-actin
β-actin

## Slide 2
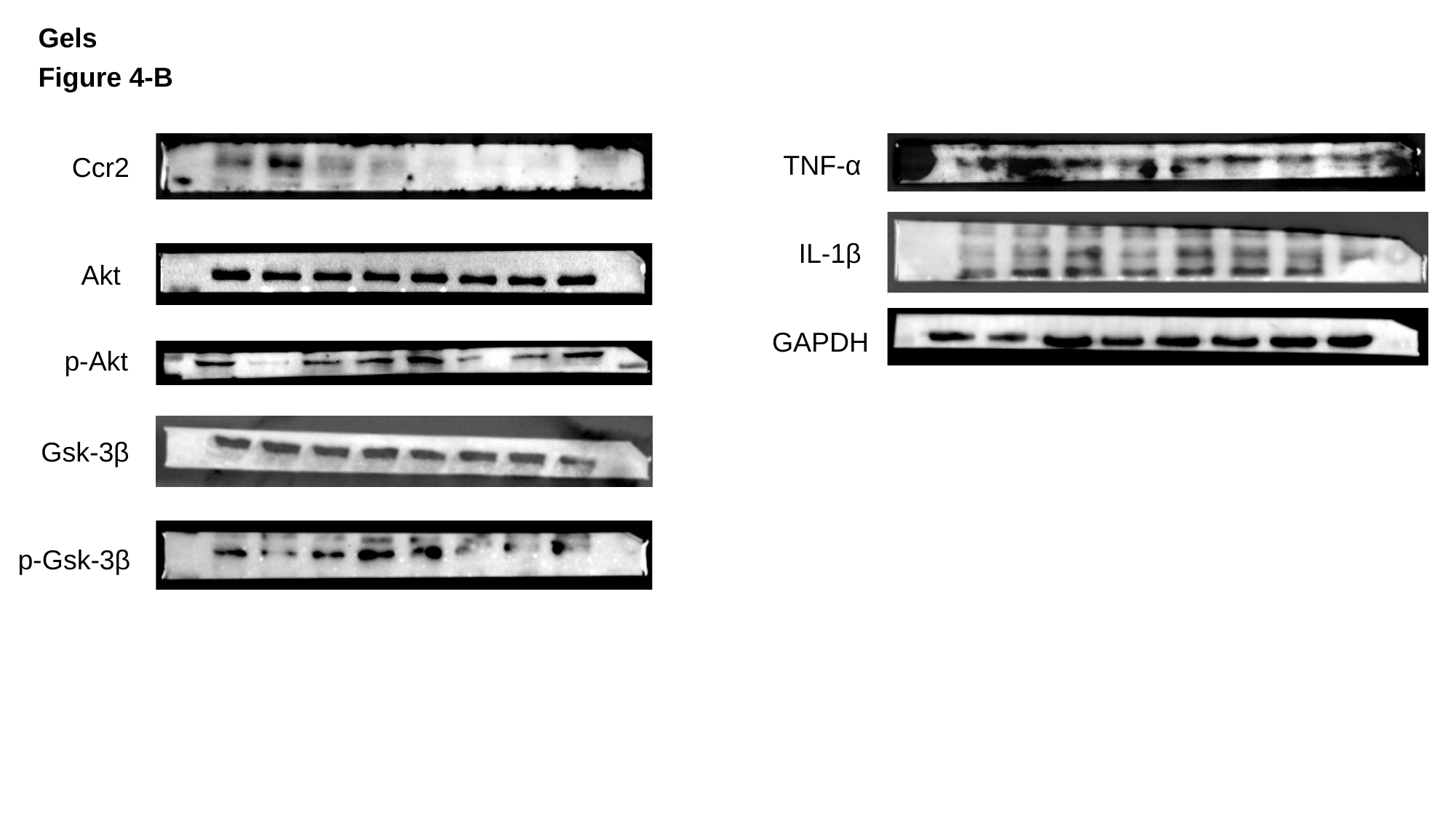

Gels
Figure 4-B
TNF-α
Ccr2
IL-1β
Akt
GAPDH
p-Akt
Gsk-3β
p-Gsk-3β

## Slide 3
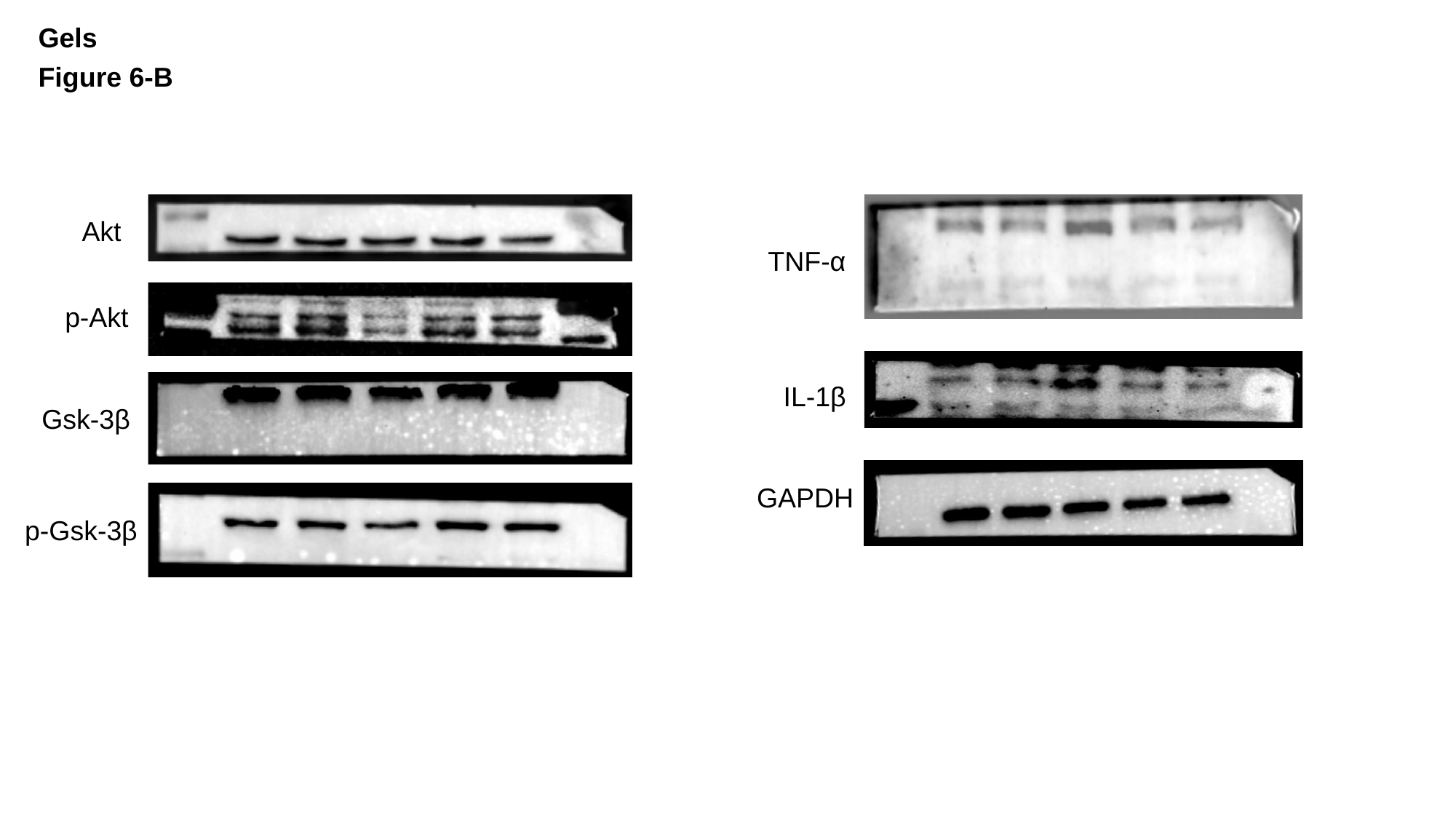

Gels
Figure 6-B
Akt
TNF-α
p-Akt
IL-1β
Gsk-3β
GAPDH
p-Gsk-3β
